# Accurate and Robust Non-rigid Point Set Registration using Student’s-t Mixture Model with Prior Probability Modeling

**DOI:** 10.1038/s41598-018-26288-6

**Published:** 2018-06-07

**Authors:** Zhiyong Zhou, Jianfei Tu, Chen Geng, Jisu Hu, Baotong Tong, Jiansong Ji, Yakang Dai

**Affiliations:** 1 Suzhou Institute of Biomedical Engineering and Technology, Chinese Academy of Sciences, Suzhou, 215163 China; 2 Lishui Central Hospital, Lishui, 323000 China

## Abstract

A new accurate and robust non-rigid point set registration method, named DSMM, is proposed for non-rigid point set registration in the presence of significant amounts of missing correspondences and outliers. The key idea of this algorithm is to consider the relationship between the point sets as random variables and model the prior probabilities via Dirichlet distribution. We assign the various prior probabilities of each point to its correspondences in the Student’s-t mixture model. We later incorporate the local spatial representation of the point sets by representing the posterior probabilities in a linear smoothing filter and get closed-form mixture proportions, leading to a computationally efficient registration algorithm comparing to other Student’s-t mixture model based methods. Finally, by introducing the hidden random variables in the Bayesian framework, we propose a general mixture model family for generalizing the mixture-model-based point set registration, where the existing methods can be considered as members of the proposed family. We evaluate DSMM and other state-of-the-art finite mixture models based point set registration algorithms on both artificial point set and various 2D and 3D point sets, where DSMM demonstrates its statistical accuracy and robustness, outperforming the competing algorithms.

## Introduction

Registration of point sets is of great importance in many computer vision tasks, such as medical image registration, image analysis, computer graphics, and pattern recognition. Many problems in these fields can be solved by point set registration algorithms operating on points or landmarks extracted from the input images. In medical image analysis, point set registration is necessary to match points or landmarks in 3D images for disease diagnosis, motion models, point-set-based image registration, image fusion, and construction of image atlases. In this paper, we focus on the registration model which is a key problem in non-rigid point set registration.

Mathematically, the point set registration problem can be described as follows. Let *X* ∈ *IR*^*D*^ and *Y* ∈ *IR*^*D*^ be two point sets to be registered, where *X* is a *D*-dimensional point set labeled as the target set, while *Y* is a *D*-dimensional point set labeled as the template set. The general approach of point set registration is to estimate a mapping *T* from *IR*^*D*^ to *IR*^*D*^, which yields the best transformation between the target set *X* and the template set *Y*.

Many algorithms have been proposed for point set registration. Iterative Closest Point (ICP) algorithm^1^ is the most popular algorithm owing to its low computation complexity. The traditional ICP algorithm defines the correspondences based on a closest neighbor principle. ICP finds a closest point *y*_*i*_ in *Y* for each point *x*_*i*_ in *X*. It subsequently estimates a transformation which best aligns *X* to *Y* by using a least-squares method. ICP iterates the cycle of correspondences establishment and alignment until it reaches the local minimum. However, the traditional ICP requires the initial position of the two point sets to be adequately close. ICP has been received a lot of attentions and several improved algorithms have been proposed^2–5^. Liu reviewed the improvements over ICP^6^.

Instead of aligning a one-to-one correspondence based on a closest distance criterion, the Robust Point Matching (RPM) algorithm^7^ proposed by Gold *et al*. and its variants^8,9^, alternatively estimate soft-assignment of correspondences and transformation, leading to allowing for fuzzy correspondences, and^9^ subsequently used Thin-Plate-Spline (TPS) to re-parameterize the transformation that resulted into the TPS-RPM algorithm. Tsin and Kanade^10^ proposed a kernel-correlation-based point set registration approach, considering the non-rigid point set registration as an alignment between two distributions. This approach parameterizes the point sets using explicit TPS parameterizations, which is equivalent to a regularization of second order derivatives of the transformation. Their algorithm attempts to align the given two point sets without explicitly estimating the correspondences, leading to a more robust algorithm against degeneration (such as missing correspondences and outliers).

Chui *et al*.^11^ pointed out that the processing of alternative correspondence estimate and transformation in the RPM algorithm is equivalent to the Expectation Maximization (EM) framework for Gaussian mixture model (GMM), in which one point set is considered as GMM centroids and the other one is considered as data^12^. GMM is a well-known mixture model, widely used to formulate non-rigid point set registration as it is a natural and simple way to describe the given point sets. Revow *et al*.^13^ represented the contour-like point sets using splines and modeled them by the probabilistic GMM formulation, where GMM centroids were uniformly positioned along the contours. This algorithm allows non-rigid transformation for point sets. Similar to^9^, Myronenko *et al*.^14^ proposed a robust point set registration framework. Myronenko *et al*.^15^ later introduced the Coherent Point Drift (CPD) algorithm, which enforced the points drift coherently by regularizing the transformation following the Motion Coherence Theory (MCT)^16,17^. The major difference between the two algorithms proposed in^9,15^ is that^9^ re-parameterizes the transformation via TPS, while^15^ re-parameterizes the transformation by using Gaussian radial basis functions (GRBF). However, the CPD algorithm aligns a same mixture proportion for all mixture components and introduce an additional uniform distribution in mixture model for improving robustness against outliers, noise and occlusion^18^. Jian and Vemuri^19^ modeled both point sets using GMM and introduced a general robust framework involving the minimization of the *L*_2_ distance between Gaussian mixtures. Tustison *et al*.^20^ also represented point sets by using a GMM with an anisotropy covariance. In addition, features such as mutual information^21^ and shape^22–24^ extracted from images or point sets are incorporated into point set registration. Wang *et al*.^25^ generalized a *L*_2_ divergence and obtained closed-form solutions for registration. Subsequently, Wang *et al*.^26^ used a similar model to simultaneously align multiple point sets. However, it is well known that the GMM-based non-rigid point set algorithms are sensitive to significant amounts of outliers and missing correspondences since they use an additional component to represent the heavy tail of the mixture model^27^.

There are also several algorithms that attempt to align two point sets using the Student’s-t mixture model (SMM) to improve the accuracy and robustness against outliers and missing correspondences. SMM has been introduced as an alternative to GMM, providing an effective and non-heuristic mean to handle degradations such as missing correspondences and outliers^28^. It is worth to point out that, mathematically, the Student’s-t distribution corresponds to a Gaussian distribution when the degree of freedom (DoF) *γ* → ∞, making the Gaussian mixture model be a special case of the Student’s-t mixture model^27^. The Student’s-t mixture model has heavily tails, leading to a natural and elegant model for modeling the given point sets with degradations^29^. Gerogiannis *et al*.^30,31^ proposed a SMM-based rigid point set registration algorithm which was more robust than the GMM-based algorithms. However, it is regretful that the proposed algorithm is limited to rigid point set registration. In previous work, we introduced a SMM-based non-rigid point set registration method (called pSMM in this paper) for contour-like and surface-like point sets^32^, subsequently, we apply it for matching surface-like points^33^. Unfortunately, pSMM utilized EM framework to directly calculate the prior probability, which is a least-square-based method for fitting parameters, whose lack of robustness is well known. Moreover, it is an arduous task to get closed-form solutions for the SMM-based non-rigid point set registration in the EM framework^34^. To overcome this problem, Peel and McLachlan considered^34^ SMM as an infinite mixture model of the scaled GMM integral form to get the closed-form solutions in EM framework^35,36^. Liu and Rubin indicated that convergence of estimating parameters of SMM in EM framework is slow, they subsequently extended the EM framework in the form of ECM and ECME algorithms^37,38^. Recently, the Student’s-t distribution and the Student’s-t mixture model also demonstrate their accuracy and robustness against outliers in various applications, such as data cluster^39,40^, data classification^41^, and image segmentation^42–44^. However, the prior distribution of SMM does not depend on the given point sets and the a same mixture proportion is assigned to all data in the existing approaches^29,31,40^. Additionally, the existing point set registration approaches do not take into account the local spatial representation of the input point sets. In order to overcome the lack of local spatial representation, Ma *et al*.^45^ introduced a novel transformation estimation method using *L*_2_*E* estimator for building robust sparse and dense correspondences. Some feature descriptors, such as shape context, are utilized for establish rough correspondences in their work. Ma *et al*.^46^ considered point set registration as the estimation of a mixture of density, where the local feature is used to assign the membership probability of the mixture model.

In this paper, we proposed a more accurate and robust non-rigid point set algorithm, called DSMM, by using Dirichlet distribution in the Student’s-t mixture model to formulate the various mixture proportion and assign them to corresponding mixture components, instead the same value in the existing methods. Comparing with the existing state-of-the-art point set registration algorithms (include pSMM), the key contributions of our work are: (1) We introduce the idea of considering the mixture component label vector as random variables, which is a major difference from the existing point set registration, where the mixture proportions are considered as discrete labels. We consequently utilize the Dirichlet distribution as a natural model for formulating the mixture proportion in the Student’s-t mixture model, and assign various mixture proportion *w*_*mn*_ for each observation *x*_*m*_ belonging to corresponding component *y*_*n*_. It is worth to point out that the main difference between DSMM and pSMM is that pSMM mathematically use a least-squared method to estimate the prior probabilities, while DSMM utilities an Dirichlet distribution for modeling it, which is detailed in subsection 2.2. (2) We further propose a general mixture model family for point set registration based on the hidden variables in the Bayesian framework, which reveals the relationship of DSMM and the existing methods in subsection 2.3. We consider the Student’s-t mixture model as infinite mixture of scaled Gaussian mixture model as Peel and McLachlan did^34^, and subsequently parameterize the hidden variables using Dirichlet distribution. (3) In order to incorporate the local spatial relationship between neighboring points, we further formulate the mixture proportions by the parameters of Dirichlet distribution by representing the posterior probabilities in a linear smoothing filter.

The rest of this paper is organized as follows. In the section 2, we present the main idea of the Dirichlet distribution for modeling the mixture proportions of the Student’s-t mixture model, and further propose a general mixture model family for point set registration, where DSMM and existing approaches can be considered as its member. Section 3 contains some qualitative and quantitative evaluations on 2D and 3D point sets with outliers and missing correspondences. Finally, we present a discussion in section 4 and a conclusion in section 5.

## Method

### Student’s-t mixture model for registration

In this section, we start with briefly reviewing our previous work on point set registration based on Student’s-t mixture model^32^. Let *X*_*M*×*D*_ = (*x*_1_, … *x*_*M*_)^*T*^ denotes a *D*-dimension point set considered as an observation, *Y*_*N*×*D*_ = (*y*_1_, … *y*_*N*_)^*T*^ denotes the other *D*-dimension point set. Each point *y*_*n*_ is considered as a component of the Student’s-t mixture model. The probability density function of the Student’s-t mixture model with *N* components is defined as

1 $$f({x}_{m}|{y}_{n},{w}_{n},{\sigma }^{2},{\gamma }_{n})=\sum _{n=1}^{N}{w}_{n}S({x}_{m}|{y}_{n},{\sigma }^{2},{\gamma }_{n})$$

where *w*_*n*_ is a prior probability (mixture proportion) for *y*_*n*_, satisfying the following constraint

2 $$0 < {w}_{n} < 1,\,\sum _{n=1}^{N}{w}_{n}=1$$

*S*(*x*_*m*_|*y*_*n*_,*σ*^2^,*γ*_*n*)_ represents a probability density of multivariate Student’s-t distribution, which takes the form

3 $$S({x}_{m}|{y}_{n},{\sigma }^{2},{\gamma }_{n})=\frac{{\rm{\Gamma }}(({\gamma }_{n}+D)/2)}{\sigma {({\gamma }_{n}\sqrt{\pi })}^{D/2}{\rm{\Gamma }}({\gamma }_{n}/2){(1+d({x}_{m},{y}_{n},{\sigma }^{2})/{\gamma }_{n})}^{(D+{\gamma }_{n})/2}}$$

In the Eq. (3), *d*(*x*_*m*_,*y*_*n*_,*σ*^2^) = (*x*_*m−*_*y*_*n*_)^*T*^(*x*_*m−*_*y*_*n*_)/*σ*^2^ is the Mahalanobis squared distance between *x*_*m*_ and *y*_*n*_, and Γ(·) is a Gamma function. In our registration method, each Student’s-t distribution *S*(*x*_*m*_|*y*_*n*_,*σ*^2^,*γ*_*n*_), which is called a component of the mixture model, has its own parameter set Θ_*n*_ = {*y*_*n*_,*σ*^2^,*γ*_*n*_} with its component centroid *y*_*n*_, variance *σ*^2^ (or precision 1/*σ*^2^) and degree of freedom *γ*_*n*_.

Mathematically, the multivariate Student’s-t distribution is equivalent to Gaussian distribution when its *γ* → ∞. The Student’s-t distribution provides a heavy-tailed model for fit the degradations such as data with longer than normal tails, outliers, and missing correspondences.

### Prior probability modeling with Dirichlet distribution

The prior probability *w*_*n*_ in the Eq. (1) represents the mixture proportions of the *n-*th component in the mixture model. Unfortunately, in the previous work^14,15,47^, the mixture proportion *w*_*n*_ is assigned to all correspondences, which is unreasonable as the observations vary in their locations. Moreover, the existing methods estimate the prior probabilities via a least-squared-based method in the EM framework, leading a well-known under-fitting problem for complex point set registration. Another limitation is that each observation is considered as an independent point to its neighbors. Therefore, these methods do not take into account the spatial correlation between the neighboring points in the decision process. In order to overcome the under-fitting problem and improve the robustness to noise, outliers and occlusion, we introduce Dirichlet distribution for modeling the prior probabilities and assign different prior probabilities between the observations and their correspondences.

Firstly, we rewrite the density function of Student’s-t mixture model at an observation *x*_*m*_, which takes the form

4 $$f({x}_{m}|{y}_{n},{w}_{n},{\sigma }^{2},{\gamma }_{n})=\sum _{n=1}^{N}{w}_{mn}S({x}_{m}|{y}_{n},{\sigma }^{2},{\gamma }_{n})$$

Specially, the parameter *w*_*mn*_ denotes the mixture proportion of the component *y*_*n*_ belonging to its correspondence *x*_*m*_.

Secondly, we introduce the hidden variables^27^ in the Bayesian approach to model the prior probabilities in our method. In the Bayesian approach, the complete-data vector, which composes of the hidden variables, is given by

5 $${v}_{c}={({y}_{1},\ldots ,{y}_{N},{z}_{1},\mathrm{...},{z}_{N},{u}_{1},\mathrm{...},{u}_{N})}^{T}$$

where the discrete label z_*n*_ = (*z*_1*n*_, …, *z*_*Mn*_)^*T*^ denotes the component label vector, which defines the relationships between *x*_*m*_ and *y*_*n*_ (*n* = 1, …, *N*; *m* = 1, …, *M*). *z*_*mn*_ is 1 or 0 depending on whether *x*_*m*_ belongs to the *n-*th component

6 $${z}_{mn}=\{\begin{array}{ll}1 & {x}_{m}\,{\rm{belongsto}}\,n \mbox{-} \mathrm{th}\,{\rm{component}}\\ 0 & {\rm{otherwise}}\end{array}$$

*u*_1_, …, *u*_*N*_ represent the hidden variables associated with the scaling weights of the covariance of the equivalent Gaussian distributions, which is defined as

7 $${u}_{n|{z}_{mn}=1}\sim {f}_{{\rm{\Gamma }}}({\gamma }_{n}/2,{\gamma }_{n}/2)$$

where *f*_Γ_(*x*) is the Gamma function. According to the Eq. (7), *u*_1_, …, *u*_*N*_ are independent variables if z_1_, …, z_*N*_ are given. Consequently, *x*_*m*_ is a random variable defined as^34^

8 $${x}_{m}{|}_{{u}_{n},{z}_{mn}=1}\sim {f}_{N}({y}_{n},{\sigma }^{2}/{u}_{n})$$

where *f*_*N*_(*y*_*n*_,*σ*^2^/*u*_*n*_) is a Gaussian distribution with the mean *y*_*n*_ and the covariance *σ*^2^/*u*_*n*_. We now focus on the hidden variable *z*_*n*_, which is considered as an independent variable in pSMM. We now consider *z*_*n*_ = (*z*_1*n*_, … *z*_*mn*_) as a probable label vector and formulate it by Dirichlet distribution and Dirichlet law^44,48,49^ for accurately modeling the prior probabilities. Dirichlet distribution is a natural and power method for modeling complex data by varying its parameters.

According to^29^, we get the conditional probability of the probability label *z*_*n*_

9 $$p({z}_{n}|{\alpha }_{n})={\int }_{0}^{1}p({z}_{n}|{\xi }_{n})p({\xi }_{n}|{\alpha }_{n})d{\xi }_{n}$$

where *ξ*_*n*_ = {*ξ*_1*n*_, …, *ξ*_*Mn*_} (*m* = 1, …, *M*) is the Dirichlet parameter in the *M*-dimensional probability simplex, satisfying 0 < *ξ*_*mn*_ < 1 and ${\sum }_{m=1}^{M}{\xi }_{mn}=1$; and *α*_*n*_ = {*α*_1n_, …, *α*_*Mn*_}, satisfying 0 < *α*_*mn*_ < 1, is the vector of the Dirichlet parameters. *p*(*z*_*n*_|*ξ*_*n*_) and *p*(*ξ*_*n*_ |*α*_*n*_) take the form of

10 $$p({z}_{n}|{\xi }_{n})=\frac{\prod _{m=1}^{M}{({\xi }_{mn})}^{{z}_{mn}}}{\prod _{m=1}^{M}({z}_{mn})!}$$

11 $$p({\xi }_{n}|{\alpha }_{n})=\frac{{\rm{\Gamma }}(\sum _{m=1}^{M}{\alpha }_{mn})}{\prod _{m=1}^{M}{\rm{\Gamma }}({\alpha }_{mn})}\prod _{n=1}^{N}{({\xi }_{mn})}^{{\alpha }_{mn}-1}$$

Combining the Eqs (9), (10) and (11), the probability label subsequently takes the form

12 $$\begin{array}{rcl}p({z}_{n}|{\alpha }_{n}) & = & {\int }_{0}^{1}\frac{\prod _{n=1}^{N}{({\xi }_{mn})}^{{z}_{mn}}}{\prod _{n=1}^{N}({z}_{mn})!}\frac{{\rm{\Gamma }}(\sum _{n=1}^{N=1}{\alpha }_{mn})}{\prod _{n=1}^{N}{\rm{\Gamma }}({\alpha }_{mn})}\prod _{n=1}^{N}{({\xi }_{mn})}^{{\alpha }_{mn}-1}d{\xi }_{n}\\  & = & \frac{1}{\prod _{n=1}^{N}({z}_{mn})!}\frac{{\rm{\Gamma }}(\sum _{n=1}^{N=1}{\alpha }_{mn})}{\prod _{n=1}^{N}\Gamma ({\alpha }_{mn})}\frac{\prod _{n=1}^{N}{\rm{\Gamma }}({\alpha }_{mn}+{z}_{mn})}{{\rm{\Gamma }}(\sum _{n=1}^{N}({\alpha }_{mn}+{z}_{mn}))}\\  &  & \times {\int }_{0}^{1}\frac{{\rm{\Gamma }}(\sum _{n=1}^{N}({\alpha }_{mn}+{z}_{mn}))}{\prod _{n=1}^{N}{\rm{\Gamma }}({\alpha }_{mn}+{z}_{mn})}\prod _{n=1}^{N}{({\xi }_{mn})}^{{\alpha }_{mn}+{z}_{mn}-1}d{\xi }_{m}\end{array}$$

According to the property of the probability density function, *p*(*ξ*_*m*_|*α*_*m*_) always satisfies the following condition

13 $${\int }_{0}^{1}p({\xi }_{m}|{\alpha }_{m})d{\xi }_{m}={\int }_{0}^{1}\frac{{\rm{\Gamma }}(\sum _{n=1}^{N}{\alpha }_{mn})}{\prod _{n=1}^{N}{\rm{\Gamma }}({\alpha }_{mn})}\prod _{n=1}^{N}{{\xi }_{mn}}^{{\alpha }_{mn}-1}d{\xi }_{m}=1$$

Utilizing the Eq. (13) to rewrite the Eq. (12), we could obtain the probability

14 $$p({z}_{m}|{\alpha }_{m})=\frac{1}{\prod _{n=1}^{N}({z}_{mn})!}\frac{{\rm{\Gamma }}(\sum _{n=1}^{N}{\alpha }_{mn})}{\prod _{n=1}^{N}{\rm{\Gamma }}({\alpha }_{mn})}\frac{\prod _{n=1}^{N}{\rm{\Gamma }}({\alpha }_{mn}+{z}_{mn})}{{\rm{\Gamma }}(\sum _{n=1}^{N}({\alpha }_{mn}+{z}_{mn}))}.$$

We now consider the condition of discrete label *z*_*mn*_ in the Eq. (6). Considering Γ(*x* + 1) = *x*Γ(*x*), the closed-form solution of prior probability *w*_*mn*_ is finally given by

15 $${w}_{mn}=p({z}_{mn}=1|{\alpha }_{m})={\alpha }_{mn}/\sum _{n=1}^{N}{\alpha }_{mn}$$

However, the components in the mixture model are still assumed to be independent identically distributed, which brings an attendant trouble that there is no neighborhood information for registration process since *x*_*n*_ is considered as an independent point to its neighbors. In order to solve the problem, we constraint the Dirichlet distribution with local spatial representation via defining parameter *α*_*mn*_ of the Dirichlet distribution as^42^

16 $${\alpha }_{mn}=\exp (\frac{\bar{\alpha }}{{N}_{n}}\sum _{{y}_{i}\in \partial {y}_{n}}p({y}_{n};{x}_{m}))=\exp (\frac{\bar{\alpha }}{{N}_{n}}\sum _{{y}_{i}\in \partial {y}_{n}}{p}_{mn})$$

where *p*_*mn*_ is a posterior probability, which is formulated as

17 $${p}_{mn}=\frac{{w}_{mn}S({x}_{m};{y}_{n},{\sigma }^{2},{\gamma }_{m})}{\sum _{n=1}^{N}{w}_{mn}S({x}_{m};{y}_{n},{\sigma }^{2},{\gamma }_{m})}$$

*N*_*n*_ stands for the number of neighbors locating in the window around the point *y*_*n*_, and *y*_*i*_ ∈ ∂*y*_*n*_ represents that *y*_*i*_ locates in the neighborhood of the given point *y*_*n*_. $\bar{\alpha }$ is a local spatial constraint coefficient of the Dirichlet distribution. *α*_*mn*_ contains the neighborhood information that makes registration has a spatial constraint. Moreover, only a parameter in the EM framework need to be calculated, not *M* × *N* parameters *α*_*mn*_ in the traditional Student’s-t distribution mixture model, leading our method to be a computationally effective algorithm. We finally accurately model the prior probability *w*_*mn*_ and incorporate the local spatial constraint in a simple way. Combining the Eqs (15) and (16), *w*_*mn*_ gets its closed-form as

18 $${w}_{mn}=\frac{\exp (\frac{\bar{\alpha }}{{N}_{n}}\sum _{{y}_{i}\in \partial {y}_{n}}{p}_{mn})}{\sum _{n=1}^{N}\exp (\frac{\bar{\alpha }}{{N}_{n}}\sum _{{y}_{i}\in \partial {y}_{n}}{p}_{mn})}$$

In order to get a solution of $\bar{\alpha }$, we separate *w*_*mn*_ from the probability density function (4) and estimate it by minimizing the negative log-likelihood function equivalently.

19 $$E(\Psi )=\sum _{n=1}^{N}{w}_{mn}S({x}_{m}|{y}_{n},{\sigma }^{2},{\gamma }_{n})=E({w}_{mn})+E({\gamma }_{n})+E({y}_{n},{\sigma }^{2})$$

We obtain the iterative solution of $\bar{\alpha }$ by minimizing *E*(*w*_*mn*_), or equivalently solve the following equation

20 $$\sum _{n=1}^{N}\,\sum _{m=1}^{M}{p}_{mn}(\frac{\sum _{{y}_{i}\in {y}_{n}}{p}_{mi}}{{N}_{n}}-\frac{\sum _{m^{\prime} =1}^{M}((\frac{1}{{N}_{n}}(\sum _{{y}_{i}\in {y}_{n}}{p}_{m^{\prime} i}))\exp (\frac{\bar{\alpha }}{{N}_{n}}\sum _{{y}_{i}\in {y}_{n}}{p}_{m^{\prime} i}))}{\sum _{m^{\prime} =1}^{M}\exp (\frac{\bar{\alpha }}{{N}_{n}}\sum _{{y}_{i}\in {y}_{n}}{p}_{m^{\prime} i})})=0$$

Comparing to the mathematical expressions in the MRF method^50^, we find a connection between the proposed Dirichlet-based spatial representation and the MRF method. The energy function *U*_MRF_ in the MRF method in^50^ may degenerate to the prior probability in the Eq. (18) of our method if *U*_MRF_ is set up as a diagonal matrix with the diagonal as −1, which implies that the Dirichlet distribution models the prior probabilities by using a spatial clustering method. A limitation of the previous methods is that they consider each point is independent to its neighbors, which results to the lack of a spatial correlation between the neighboring points.

The parameter set of non-rigid point set registration is defined as Ψ = (*w*_1_, …, *w*_*n*_, *γ*_1_, …, *γ*_*n*_, *y*_1_, …, *y*_*n*_, *σ*^2^), where *w*_*n*_ = (*w*_1*n*_, …, *w*_*Mn*_) represents the prior probability, whose solution has been discussed above. We subsequently separate parameters of SMM and estimate them by maximizing their log-likelihood, or by minimizing the negative log-likelihood function equivalently for calculating other parameters in the EM method. We now briefly reviews the solution of these parameter, which is detailed in our previous work^32,33^. Firstly, we consider the Eq. (19), *u*_*mn*_ can be calculated from the equation

21 $${u}_{mn}={E}_{\Psi }({u}_{n};{x}_{m},{z}_{mn}=1)=\frac{{\gamma }_{n}+D}{{\gamma }_{n}+d({x}_{m},{y}_{n},{\sigma }^{2})}$$

The solution of *γ*_*n*_ of *k*-iteration could be obtained by minimizing *E*(*γ*_*n*_). The iteration of *γ*_*n*_ is given by

22 $$-\phi (\frac{{\gamma }_{n}}{2})+\,\mathrm{ln}\,\frac{{\gamma }_{n}}{2}+1+\frac{\sum _{n=1}^{M}\,{p}_{mn}(\mathrm{ln}\,{u}_{mn}-{u}_{mn})}{\sum _{n=1}^{N}\,{p}_{mn}}+\phi (\frac{{\gamma }_{n}^{(k-1)}+D}{2})-\,\mathrm{ln}\,\frac{{\gamma }_{n}^{(k-1)}+D}{2}=0$$

where *γ*^(*k*−1)^ is an optimization solution in (*k*−1)-iteration.

Finally, we calculate the transformation field *Y*^15^ as *Y*^(*k*)^ = *Y*^(*k−*1)^ + *GW*^(*k*)^, where *G*_*M*×*M*_ is a Gaussian kernel matrix with it element *g*_*ij*_ = exp(−|*y*_*i*_−*y*_*j*_|/(2*β*)^2^) in order to reduce the oscillating energy at high frequency. *β* is a width of smoothing Gaussian filter, defining the model of the smoothness regularization. *G*(*m*;) is the column vector of the kernel matrix *G*_*M*×*M*_, and *W*_*M*×*D*_ is the weight matrix of *G*_*M*×*M*_. Using ∂*E*(*y*_*n*_, *σ*^2^)/∂*W* = 0, *W* is given by

23 $$W={(diag(\hat{P}{\boldsymbol{1}})G+\lambda {({\sigma }^{2})}^{(k-1)}I)}^{-1}(\hat{P}X-diag(\hat{P}{\boldsymbol{1}}){Y}^{(k-1)})$$

where $\hat{P}$ is a *M* × *N* matrix with its element ${\hat{p}}_{mn}={p}_{mn}{u}_{mn}$, denoting the posterior probability density corrected by *u*_*mn*_. ***1*** is a column vector of all ones; *I* is an identity matrix; *diag*(·) denotes a diagonal matrix. *λ* represents the trade-off between the goodness of maximum likelihood fit and regularization. Using ∂*E*(*y*_*n*_,*σ*^2^)/∂(*σ*^2^) = 0, *σ*^2^ is formulated as

24 $${\sigma }^{2}=\frac{\sum _{n=1}^{N}\,\sum _{m=1}^{M}\,{p}_{mn}{u}_{mn}{\Vert {x}_{m}-{y}_{n}-G(m,\bullet )W\Vert }^{2}}{D\sum _{n=1}^{N}\sum _{m=1}^{M}\,{p}_{mn}{u}_{mn}}$$

Generally speaking, the main advantages of our method are: (1) We model the prior probabilities by Dirichlet distribution in EM framework, which more accurately represents the mixture proportion of each component in the mixture model, leading an excellent method for degenerated point set registration. In the existing methods (includes pSMM), the prior probability *w*_*m*_ is directly estimated as ${w}_{m}={\sum }_{n=1}^{N}\,{p}_{mn}/N$ in M-step of EM method. Mathematically, it is a least-squared solution, which gives a point estimate to the prior probabilities and disregards the remaining uncertainty in the estimation. Therefore, low robustness and under-fitting problem are introduced into the process of optimization. Dirichlet distribution and its mixture model could automatically determine the number of necessary mixture components based on the data^51^. In DSMM, we introduce the Dirichlet distribution for modeling the prior probabilities, and then assign various mixture proportions (prior probabilities) *w*_*mn*_ of *n*-th component to its *m*-th correspondence. Rather than taking a point estimate, we model the prior probabilities using Dirichlet distribution, where Dirichlet distribution gives the posterior probability distribution over all model parameters in E-step of (*k* + 1) iteration by using the observed data together with the prior distributions. Subsequently, we utility these posterior probability distributions to estimate the prior probabilities in M-step. In general, comparing to a least-square-based estimation, the estimate of the prior probabilities via Dirichlet distribution could yield a robust and stable result, by including the resulting uncertainty into the estimation. (2) We incorporate the local spatial relationship between neighboring points into the Dirichlet distribution parameters in a simple and natural way by representing their posterior probabilities in a linear smoothing filter, leading to taking into consideration of the spatial correction in the registration process. Furthermore, it potentially supplies a universal approach to incorporate more ingenious filters for local spatial representation in the mixture model^52^.

**Figure 1:**
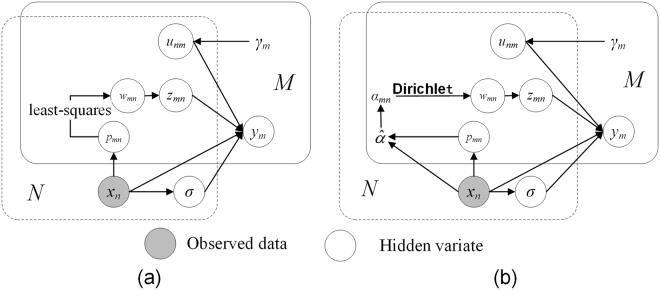
Representation of the Student’s-t mixture model with Dirichlet distribution as a directed graphical model. The *M*-box denotes the *M* observations *x*_*m*_, and the *N*-box denotes the *N* mixture components. Note that the random variable *u*_*mn*_, *w*_*mn*,_ and *z*_*mn*_ belong to both *M*-box and *N*-box, indicating that they are corresponding random variables for the Student’s-t mixture components and the observations. (**a**) pSMM for point set registration, (**b**) DSMM for point set registration.

In order to summarize the proposed method and theoretically reveal the differences between DSMM and pSMM, we represent the joint distribution of all random variables in our method via a directed graph model, as show in Fig. 1. Moreover, we will further quantitatively estimate performance of DSMM, pSMM and other competitive method in the following experiments, which will more intuitively reflect the power of modeling prior probabilities via Dirichlet distribution.

### Family of the mixture-model-based registration

We tooe an interesting observation that the mixture-model-based registration methods (included the proposed method) can be generally modeled as infinite Gaussian mixture models at a single observation *x* for potential outliers or data with longer than normal tails, which takes the form

25 $$\int {f}_{N}(x|y,{\sigma }^{{\rm{2}}}/u)dH(u).$$

Now considering an *N*-component mixture for point set registration, a general mixture model family for registration is given by

26 $$f({x}_{m}|{y}_{n},\Psi )=\sum _{m=1}^{M}{w}_{mn}(\int {f}_{N}({x}_{m}|{y}_{n},{\sigma }^{2}/{u}_{n})dH({u}_{n}))$$

where *f*_*N*_ is a general symbol for denoting a probability density function of Gaussian.

We now assume that *H* is a chi-squared distribution with the degree of freedom *γ*_*n*_ and its random variable *u*_*n*_~(*u*|*α*,*β*) = *α*^*β*^*u*^*α*^*e−*^*βu*^, where ${G}$(*u*|*α*,*β*) is a symbol of Gamma distribution. In our method, we choose *α* = *β* = *γ*_*n*_/2. According to^27,34^, it is obvious that we can rewrite Student’s-t distribution as an infinite mixture of scaled Gaussian mixture model. Therefore, we conclude that the Student’s-t mixture model is a member of the general mixture model family.

We subsequently simplify the infinite mixture to a finite mixture with two different components by placing the mass *ε* at the point *u*_*n*_ = 1 and mass (1−*ε*) at the point *u*_*n*_ = 1/*c*. The Eq. (25) therefore transforms to a Gaussian scaled mixture that takes the form as

27 $$\varepsilon {f}_{N}^{1}({x}_{m}|{y}_{n},c{\sigma }^{2})+(1-\varepsilon ){f}_{N}^{2}({x}_{m}|{y}_{n},{\sigma }^{2})$$

where *f*_*N*_(*x*|*y*,*σ*^2^) denotes the Gaussian distribution with its mean *y* and variance *σ*^2^; *ε* is a small value, representing the small proportion of observation in the mixture and *c* is a relatively large value for representing the potential degeneration that has a relatively large variance. In the two components mixture, the first term denotes the probability density of potential degeneration, while the second term denotes the probability density of normal data. Comparing to the Student’s-t mixture model, the major limitation of Gaussian scaled mixture is lack of robustness to degeneration due to its additional Gaussian components to capture the tail of the distribution, as shown in the Eq. (27).

We further simplify the Gaussian scaled mixture model. We now assume that *ϕ*_1_ is a uniform distribution, which is given by *ϕ*_1_ = *N*/*M*; *ϕ*_2_ is a Gaussian distribution, and simultaneously fix *w*_*mn*_ as a constant, satisfying *w*_*mn*_ = 1/*M*. The Eq. (26) finally transforms to *ε*/*N* + (1−*ε*)(*f*_*N*_(*x*|*y*,*σ*^2^))/*M*, which takes the same form as CPD. It is obviously to find that CPD is a member of the large family, which is formulated by the Eq. (26). Moreover, it is worthy to point out that RPM-based registration methods, such as RPM-TPS and RPM-RBF are mathematically equivalent to CPD in the EM framework, which leads RPM-based methods to be members of the mixture model family. Theoretically, the discrete latent variable *z*_*mn*_ specifies which component of the Student’s-t mixture model generates the observation *x*_*n*_, and the continuous latent variable *u*_*mn*_ specifies the scaling of the corresponding equivalent Gaussian distribution. Consequently, pSMM will transform to CPD if *z*_*mn*_ = 1, *u*_*mn*_ = 1, and *γ*_*n*_→∞ simultaneously. The degrees of freedom *γ*_*n*_ is a trade-off between robustness and efficiency. A small DoF *γ*_*n*_ can appropriately assign a small weight to the outliers or missing correspondences depending on the input data, while a relative larger value of DoF tends to fit a Gaussian mixture model to the data. Actually, the degree of freedom reflects the assumption on the amount of noise in the point sets, which plays an important role in point matching. For the initialization of the degrees of freedom, we always use the value 1 (multivariate Student’s-t distribution reduces to Cauchy distribution when *γ* = 1) to maximize the robustness at the beginning of registration process.

In the existing methods, the major disadvantage is that the parameter *z*_*mn*_ is considered as a discrete label *z*_*m*n_ = {0,1}. Another limitation of the existing mixture-model-based method is their under-fitting for prior probabilities. It is easily understood by recalling the maximization of prior probabilities in the EM framework. The estimation of prior probabilities in these methods mathematically is a least-square solution, leading to a well-known under-fitting problem. In order to get a more precise model, we consider the label *z*_*mn*_ as a random variable following a multinomial distribution with its probability vector *ξ*_*n*_ = {*ξ*_1*n*_, … *ξ*_*Mn*_}. According to the multinomial definition, the conditional distribution takes the form as

28 $$p({z}_{n}|{\xi }_{n})=\frac{K!}{\prod _{m=1}^{M}({z}_{mn})!}\prod _{m=1}^{M}{({\xi }_{mn})}^{{z}_{mn}}$$

where *ξ*_*mn*_ > 0 and ∑_*n*_*ξ*_*mn*_ = 1. The multinomial model represents the probability *ξ*_*mn*_ of observation *x*_*m*_ belonging to the component *y*_*n*_ with *K* realizations, satisfying $K={\sum }_{m=1}^{M}{z}_{mn}$. When the multinomial distribution is used to generate the correspondences, the distribution of the number of emissions (i.e., counts) of an individual component follows a binomial law^53,54^

29 $$p({z}_{mn}|{\xi }_{mn})=(\begin{array}{c}K\\ {z}_{mn}\end{array}){({\xi }_{mn})}^{{z}_{mn}}{(1-{\xi }_{mn})}^{K-{z}_{mn}}.$$

The above equation reveals that it is a small probability to a point corresponding to multi-component under the multinomial model, since the count of a single point corresponding to components decays exponentially. A better approach is hierarchical: the probabilities of correspondences between point *x*_*m*_ and component *y*_*n*_ is generated by multinomial, whose parameters are formulated by Dirichlet distribution, which is also called Dirichlet compound multinomial^55^. As discussed in the subsection 2.2, we finally formulate the mixture proportion by using parameters of Dirichlet distribution. Jian *et al*.^19^ revealed the relationship between point set registration methods from the view of the divergence function.

Generally, we generalize a family of mixture-model-based point set registration from the view of hidden variables in the Bayesian framework, and summarize a relationship between DSMM and the existing mixture-model methods in the Table 1.

**Table 1:** Relationship between the general family of mixture model registration and the existing methods.

$${\sum }_{{\boldsymbol{m}}{\boldsymbol{=}}1}^{{\boldsymbol{M}}}{{\boldsymbol{w}}}_{{\boldsymbol{mn}}}(\int {\boldsymbol{\varphi }}({{\boldsymbol{x}}}_{{\boldsymbol{m}}}|{{\boldsymbol{y}}}_{{\boldsymbol{n}}}{\boldsymbol{,}}{{\boldsymbol{\sigma }}}^{{\bf{2}}}/{{\boldsymbol{u}}}_{{\boldsymbol{n}}}){\boldsymbol{dH}}({{\boldsymbol{u}}}_{{\boldsymbol{n}}}))$$				
	*w*_*mn*_	*ϕ*	*H*	*u*
DSMM	*p*(*z*_*mn*_ = 1|*ξ*_*n*_)*p*(*ξ*_*n*_|*α*_*n*_)	Gaussian mixture	chi-squared	*u*~(*u*|*γ*_*n*_/2, *γ*_*n*_/2)
SMM	*p*(*z*_*mn*_ = 1|*x*_*m*_) = {0,1}	Gaussian mixture	chi-squared	*u*~(*u*|*γ*_*n*_/2, *γ*_*n*_/2)
PR-GLS	*τ*/|*I*| or (1−*τ*)/(*N*−*I*)	Gaussian mixture	discrete value	constant
CPD	1/*M*	Gaussian mixture	discrete value	constant
RPM-RBF	1/*M*	GRBF	discrete value	constant
RPM-TPS	1/*M*	thin plate spline	discrete value	constant

### Data availability statement

All data was obtained from public data collections, including dir-lab (https://www.dir-lab.com/index.html) and ADNI(http://www.adni-info.org/), all these database allow researches reproduce their images and data.

### Ethical approval

All data used in our experiments are from public image bases, and permit researches use images for algorithm research. All clinical data has been approved by the Medical Ethics Committee of Lishui Central Hospital and Suzhou Institute of Biomedical Engineering and Technology, Chinese Academy of Science, and has been allowed for carrying out experiments.

## Results

In this section, we qualitatively and quantitatively evaluate DSMM on various point sets, such as artificial data, points extracted from various medical images, and points form surface scan models. These point sets have various shapes, including 2D contour-like point sets, 3D cloud-like and surface-like point sets. In order to show the performance of our method, we compare DSMM with other state-of-the-art non-rigid point set registration (PR-GLS^46^, pSMM^32^, GMM-L2^19^, CPD^15^, RPM-TPS^9^, and its variety RPM-RBF) in the following evaluations. The performance of DSMM and pSMM will be intuitively shown in these evaluations. It is worth to point out that we directly perform DSMM on all point sets without any preprocessing (including rigid registration initialization), except data normalization. We only simply set *β* = 2, initial value of DoF *γ* = 1 in all tests, which is also a reflection of the robustness of DSMM.

### Qualitative evaluations

**Figure 2:**
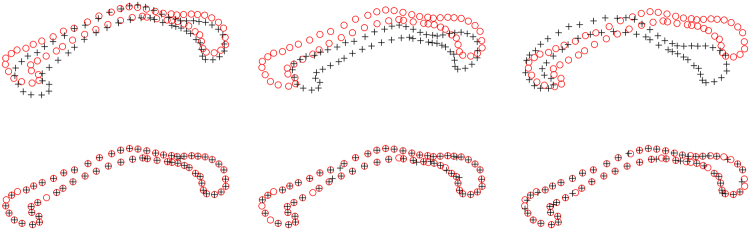
Performance of DSMM on 2D Corpus Callosum data. The target points are denoted as red “o” and the template points are denoted as black “+”. Each point set contains 63 points respectively. We align the black “+” to the red “o” by using DSMM. The point sets before registration is superimposed in the top row, and the performance of DSMM is shown in the bottom row.

We firstly demonstrate the qualitative evaluation of DSMM on 2D contour-like point sets. Specifically, Fig. 2 shows three examples of 2D contour-like Corpus Callosum (CC), which are from http://www.nitrc.org/. Each point set contains 63 points extracted from outer contour of CC in brain MR images of several normal subjects. The top row in Fig. 2 shows three pairs of Corpus Callosum point sets before registration and figures on bottom row show the performance of DSMM.

**Figure 3:**
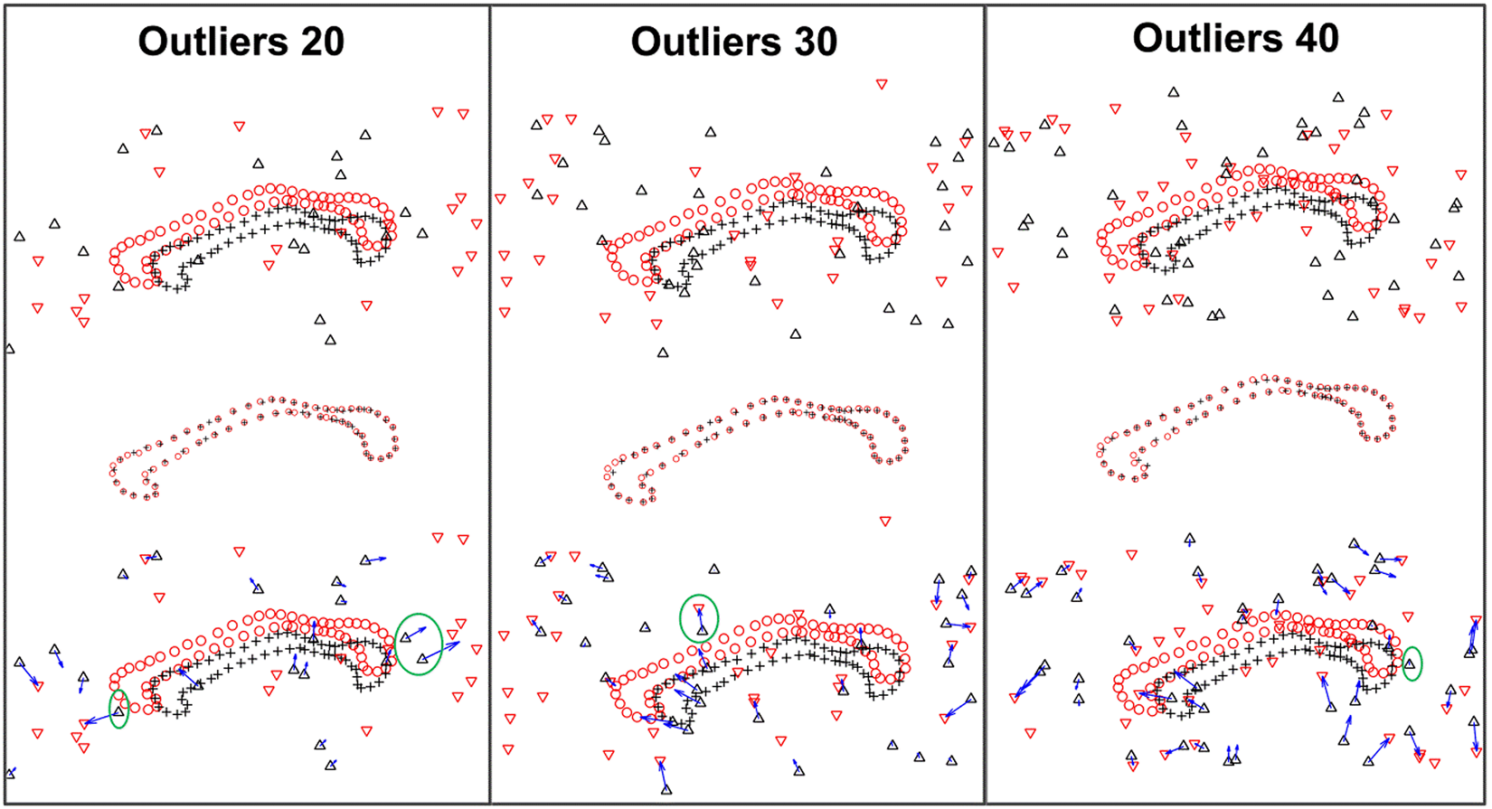
Performance of DSMM on 2D Corpus Callosum data with outliers. The data has been break up one-to-one correspondence by adding different number of additional uniform distribution outliers both in the template set and the target set. In the top row, the red “o” represents the data points in the target set, and the black “+” represents the correspondence in the template sets. For clarity, we denote outliers in the target set with red “∇”, and the outliers in the template set with black “∆”. The figures of middle row show the transformations that map all data points in the template set to their correspondences, resisting the influence of the outliers in the target set. In the bottom row, we overlap the transformations of outliers on the template set on the degenerated points before alignment, demonstrating that our method has an ability to handle most outlier, except few points very closed to the black “+”. The results intuitively show that DSMM is accurate and robust against significant number of outliers.

We add various numbers of additional random outliers with uniform distribution. Examples of such point sets (with additional 32%, 48% and 63% outliers) are respectively shown in the top row of Fig. 3. The middle row shows the final registration results, which demonstrates the data points accurately match to their correspondences, resisting the impaction of the outliers. In order to intuitively show the displacement vector of outliers, we overlap the warp of outlier on the point sets before registration, which demonstrates the transformation maps the most outliers to the sound positions, except few points who are much closed to the data points.

### Quantitative evaluations

We perform quantitative evaluations on 2D counter-like datasets, 3D cloud-like and 3D surface-like datasets for DSMM and other competing non-rigid point set registration algorithms. To take quantitative evaluations, we use the mean 3D Euclidean magnitude distance and standard deviation between correspondences as a statistical measure. In the quantitative evaluations, we show the performance of DSMM, PR-GLS^46^, pSMM^32^, GMM-L2^19^, CPD^15^, RPM-TPS^9^, and its variety RPM-RBF. Comparing to the existing methods, the major difference of our method is that DSMM models the prior probabilities by using Dirichlet distribution and assigns the various prior probability values for components, while the existing methods estimate a prior probability by a least-squared solution. PR-GLS assigns the membership probability *w*_*mn*_ based on shape context feature, so that the local structure information can also be used to achieve good performance.

**Figure 4:**
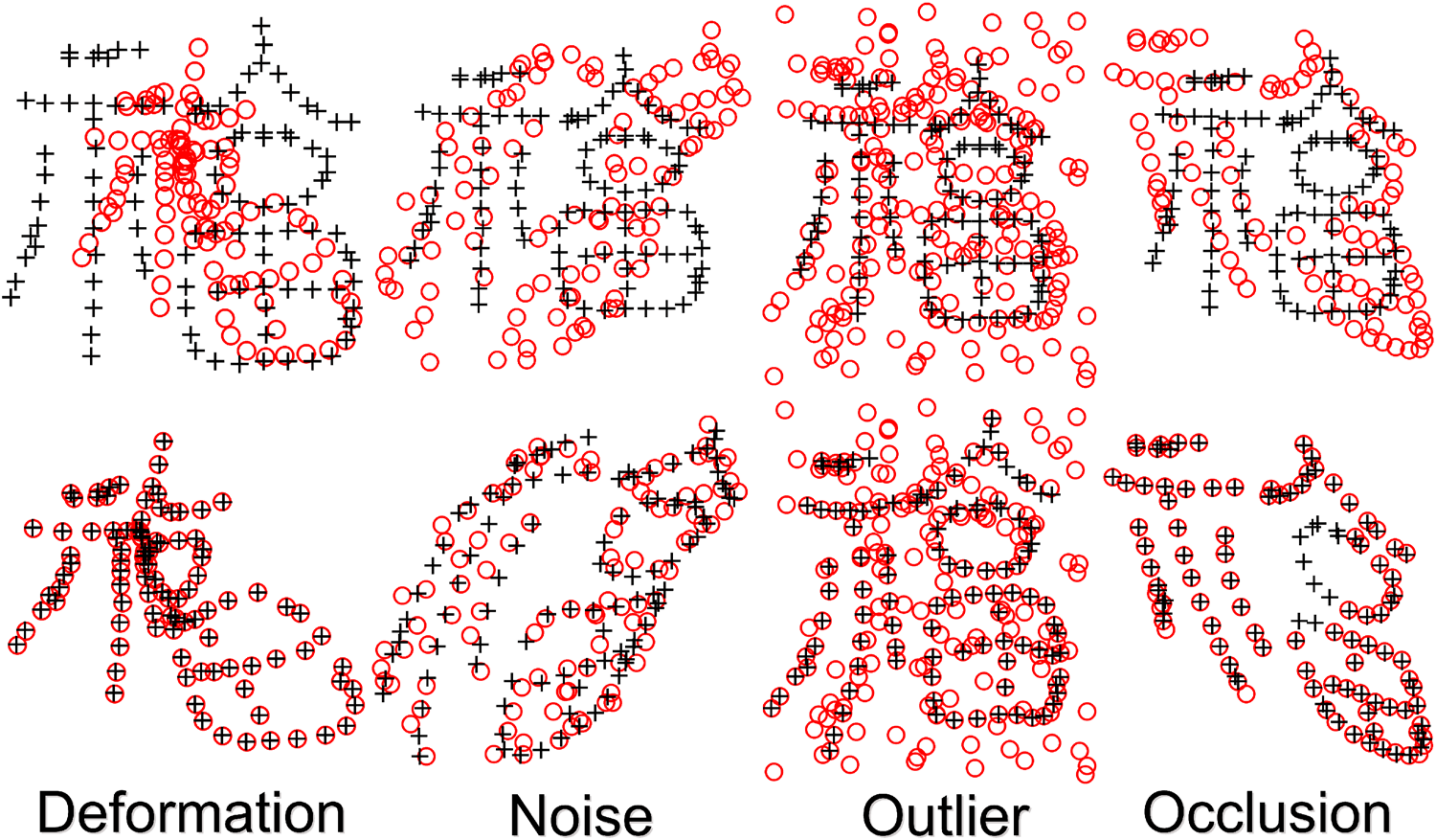
Performance of DSMM on 2D Chinese character shapes with deformation, noise, outlier and occlusion. We align the template point set (black “+”) to the target set (red “o”). For each sample, the figures in the top row show superimposed points of template point set and the target data before registration, and the figures in bottom show the registration results.

**Figure 5:**
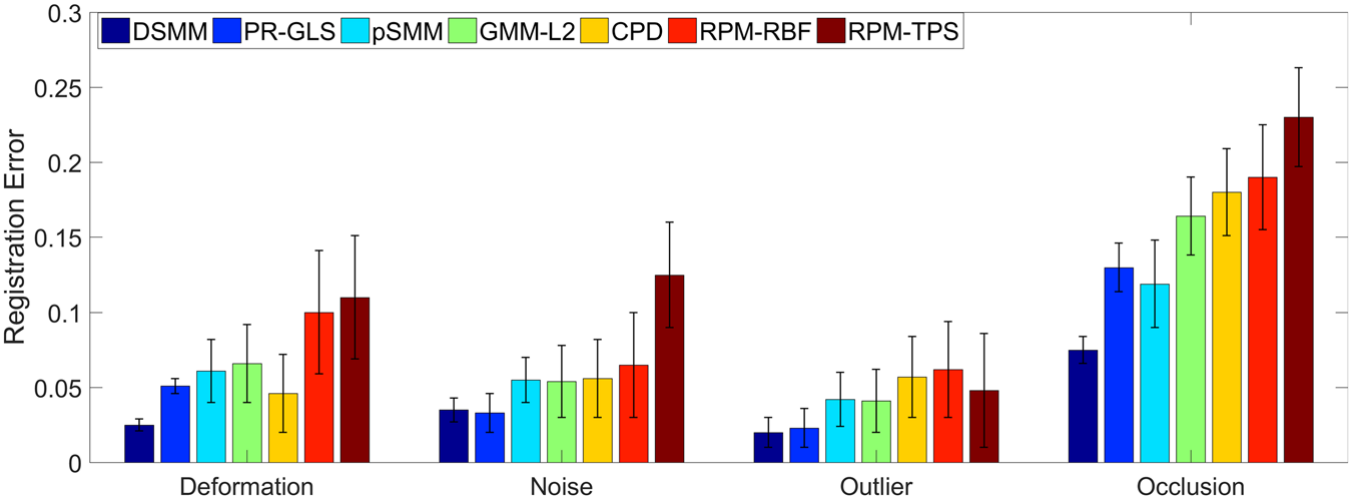
Comparison of DSMM with PR-GLS, SMM, GMM-L2, CPD, RPM-RBF and RPM-TPS on Chinese characters with deformation, noise outliers and occlusion. The y axis of error bar indicates the statistical registration error, where a small value reveals a good performance.

We perform the first quantitative evaluation on 2D Chinese characters^46^ with deformation, noise, outliers and occlusion (the ratio of noise, outliers, and occlusion is from 10 to 50%). Each point set contains 105 normal points. The superimposed points of Chinese character are respectively shown in the top row of Fig. 4. The goal of our experiments are to align the template points (black “+”) to their correspondences in the red point set (red “o”). The performance of DSMM seems good, which accurately and robustly matches the correspondences. The registration results are intuitively shown in the bottom of Fig. 4. Figure 5 shows the statistical registration results of DSMM and other completing methods. The y axis of bar in Fig. 5 indicates the mean registration error of each method, where a small error value indicates a good performance. We break the one-to-one correspondence by add noise, outliers, and removing points in these datasets. Benefitting from Dirichlet distribution and Student’s-t mixture model, the statistical results show that DSMM performs the best results, which are slightly better than PR-GLS and significantly better than other five methods.

**Figure 6:**
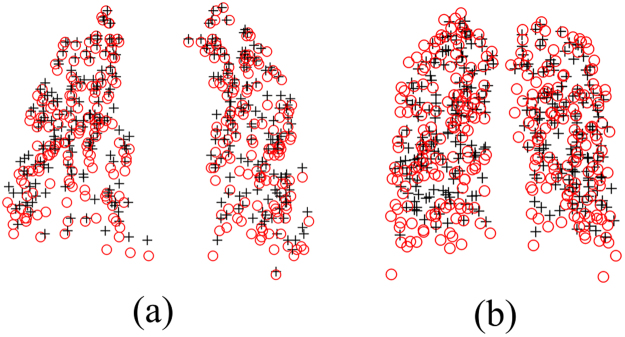
Examples of 3D cloud-like point sets extracted from thoracic 4D CT images. Each subject contains a pair of 3D lung point sets with 300 points both in template set and target set respectively. The target point sets are extracted from images of the maximum inhalation phase and the template point set are extracted from images of the maximum exhalation phase. We denote the target points with red “o” and the template ones black “+”. (**a)** An example of the 4D CT lung dataset used in the quantitative evaluation. (**b**) An example of the COPD dataset.

**Figure 7:**
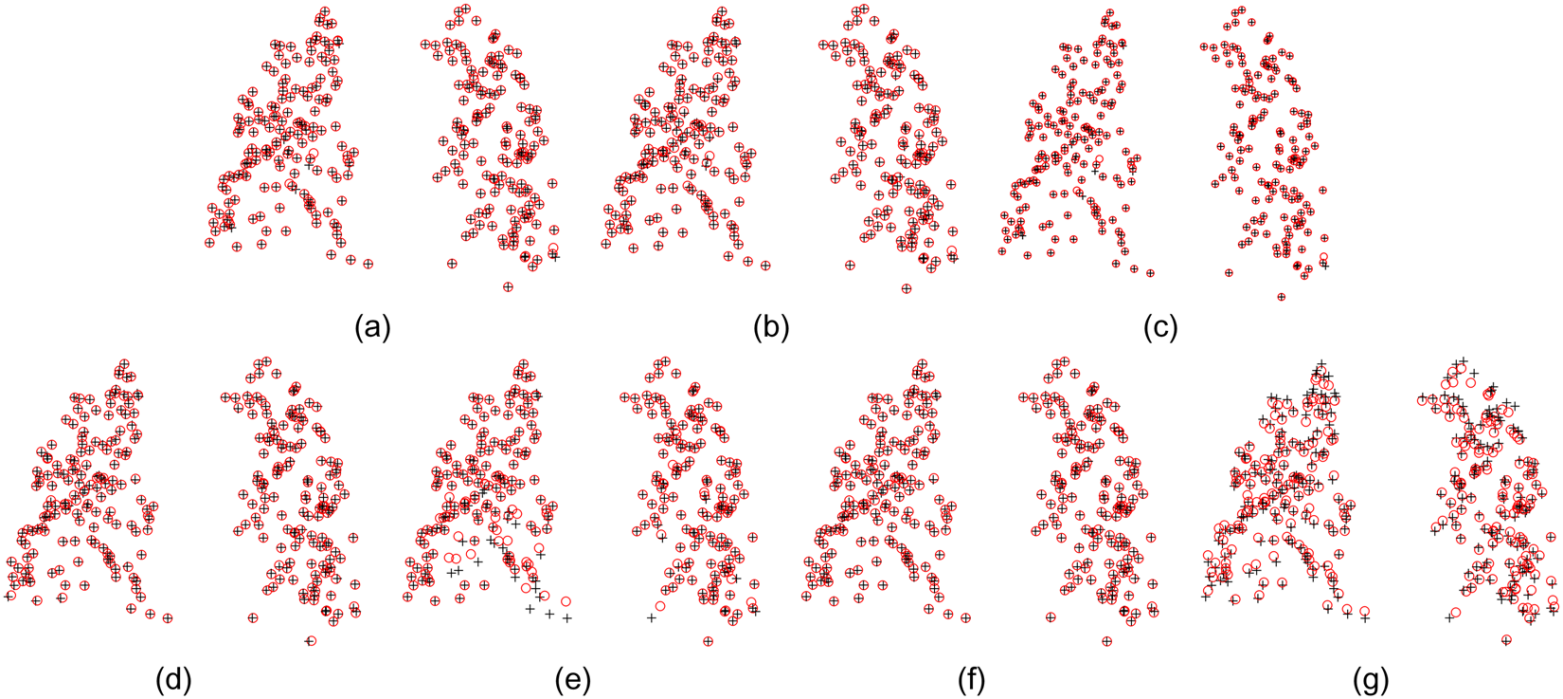
Performance of non-rigid point set registration algorithms on the point set extracted from 4D CT, which is shown in Fig. 6(a). (**a**) DSMM, (**b**) PR-GLS, (**c**) pSMM, (**d**) GMM-L2, (**e**) CPD, (**f)** RBF-RPM, (**g**) TPS-RPM.

**Figure 8:**
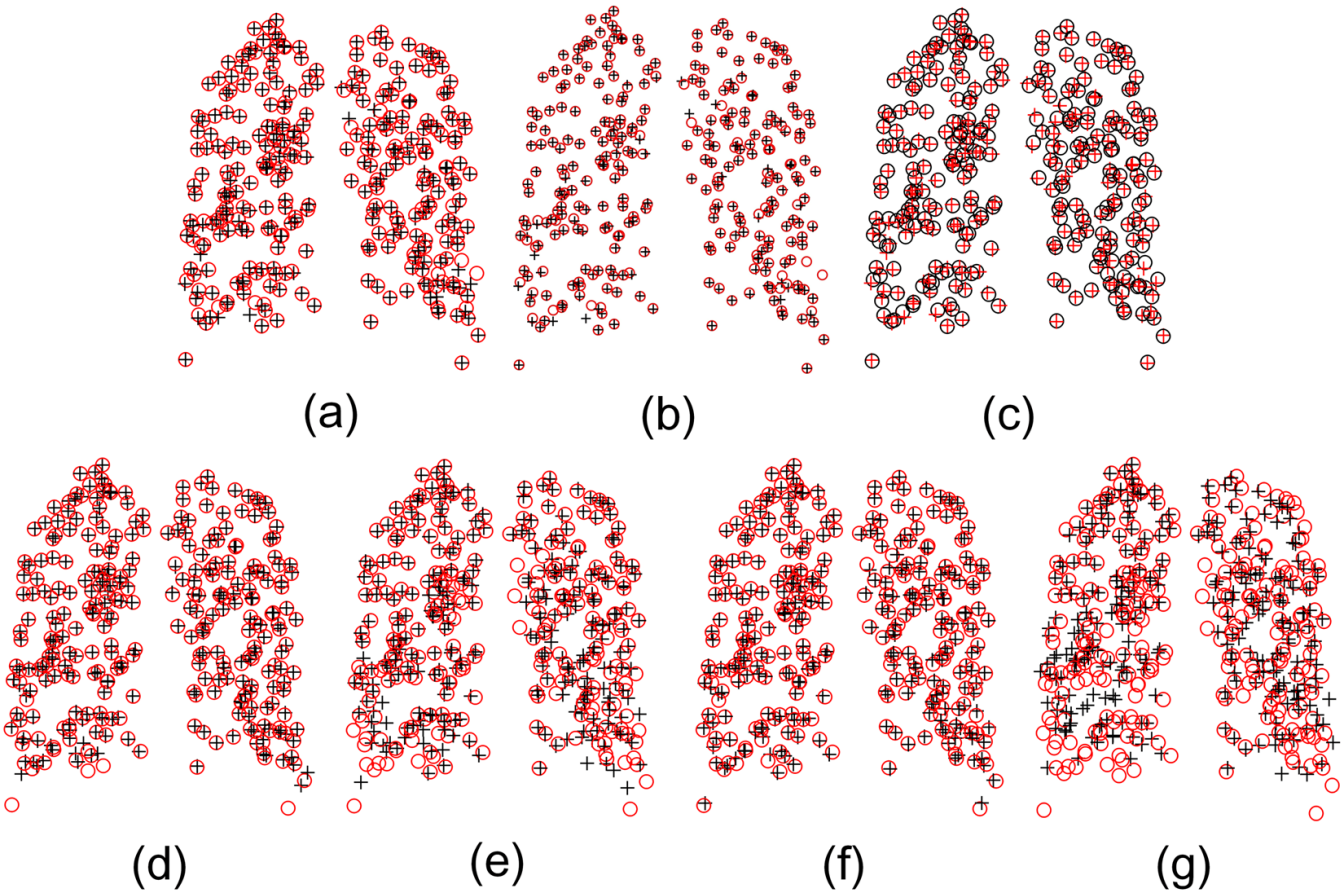
Performance of non-rigid point set registration algorithms on the point set extracted from COPD, which is shown in Fig. 6(b). (**a**–**f**) show the final registration results of DSMM, PR-GLS, pSMM, GMM-L2, CPD, RBF-RPM, and TPS-RPM respectively.

**Figure 9:**
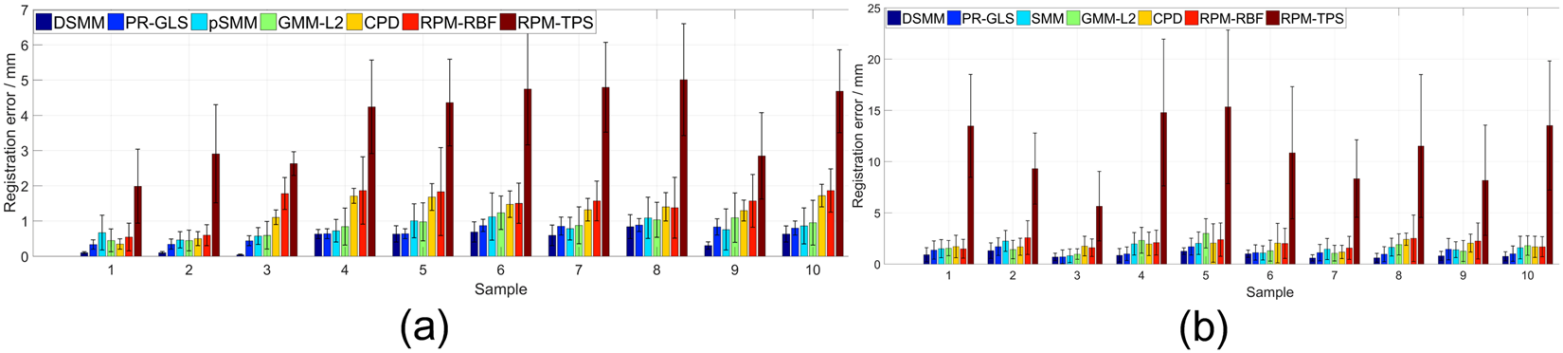
Quantitative evaluations of non-rigid point set registration algorithms on twenty 3D lung point sets, in which 10 samples are extracted from 4D CT and the other 10 samples are extracted from COPD. Mean 3D Euclidean magnitude distance between correspondences is used as a statistical measure for quantitative evaluations. The mean distance of each sample before registration is demonstrated in Table 3. (**a**) Performance of the non-rigid point set registration algorithms on 3D point sets extracted from thoracic 4D CT images. (**b**) Performance of each algorithm on the dataset extracted from COPD images. DSMM outperforms other competing algorithms both on the datasets from thoracic 4D CT images and COPD images.

**Figure 10:**
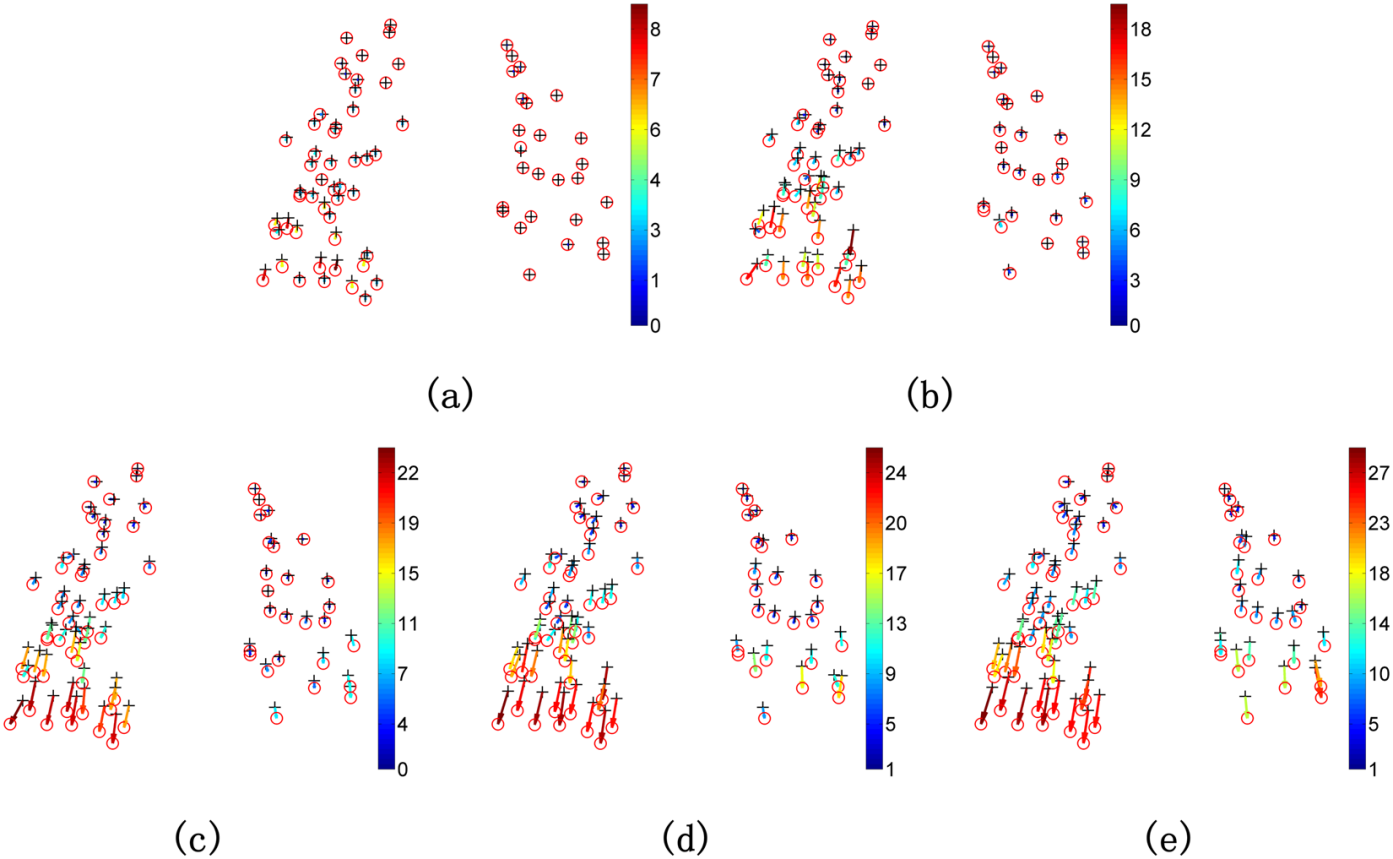
Performance of DSMM on a 4D point set from 4D CT for thoracic motion modeling. 75 points are identified from each expiratory phase image (T00, T10, T20, T30, T40 and T50), where T00 are from the maximum inhalation phase, and T50 are from the exhalation phase image. We also denote the points from the maximum inhalation phase with red “o” and points from other phase with black “+”. (**a**) Performance of DSMM on a point set between T00 and T10, (**b**) T00 and T20, (**c**) T00 and T30, (**d**) T00 and T40, (**e**) T00 and T50.

The second quantitative evaluation is performed on 20 samples of real 3D cloud-like lung datasets with 10 point sets extracted from thoracic 4D CT images^55^,^56^ and the other 10 point sets extracted from COPD images^56^, shown in Fig. 6. Each sample has a pair of 3D lung point set, one is identified from the maximum inhalation phase image and the other is identified from the maximum exhalation phase image. Each 3D lung point set respectively has 300 points, which are selected by experts to make the two point sets correspond to each other. It is a herculean task for non-rigid registration algorithms to match cloud-like point sets accurately due to lack of topological structures or geometry structures in such data. Table 2 demonstrates the mean 3D Euclidean distance between correspondences for each point set before registration. Figures 7 and 8 respectively show the performance of DSMM and other non-rigid point set algorithms on samples of datasets from 4D CT and COPD. From the statistical measure shown in Figs 9, 10 and Table 3, we can intuitively see the performance of DSMM is better than other mixture-model-based algorithms on real 3D cloud-like point sets.

**Table 2:** Mean 3D Euclidean magnitude distance and standard deviation (unit: mm) for all subjects of 3D point sets before registration.

	1	2	3	4	5	6	7	8	9	10
4D CT	4.01 ± 2.91	4.65 ± 4.09	6.73 ± 4.21	9.42 ± 4.81	7.10 ± 5.14	11.10 ± 6.98	11.59 ± 7.87	15.26 ± 9.11	7.82 ± 3.99	7.63 ± 6.54
COPD	25.90 ± 11.57	21.77 ± 6.46	12.29 ± 6.39	30.90 ± 13.49	30.90 ± 14.05	28.32 ± 9.20	21.66 ± 7.66	25. 57 ± 13.61	14.84 ± 10.01	22.48 ± 10.64

**Table 3:** Mean 3D Euclidean magnitude distance (unit: mm) for all 3D point sets after registration.

	DSMM	PR-GLS	pSMM	GMM-L2	CPD	RBF-RPM	TPS-RPM	before warp
4D CT	**0.455** ± **0.169**	0.685 ± 0.173	0.792 ± 0.444	0.849 ± 0.490	1.205 ± 0.285	1.449 ± 0.671	3.620 ± 1.219	8.52 ± 5.56
COPD	**0.917** ± **0.4766**	1.326 ± 0.7966	1.576 ± 0.9214	1.660 ± 0.9630	1.852 ± 1.0993	2.017 ± 1.3862	11.099 ± 5.5439	23.46 ± 10.31

In order to evaluate the performance of our method on the various distortion, we then perform the third quantitative evaluation on 4D CT point sets identified from thoracic 4D CT images^56^. Each 4D CT point set consists of six expiratory phases (T00, T10, T20, T30, T40 and T50) and there are 75 points (a subset of the point set containing 300 points) in each sample. The T00 point sets are identified from the maximum inhalation phase images, and the T50 point sets are identified from the maximum exhalation phase images. The T10, T20, T30, and T40 point sets are respectively extracted from the expiratory phase images between the maximum exhalation phase and the maximum exhalation phase. As shown in Fig. 10, the red “o” denotes the point in T00 image, the black + denotes the point in T10~T50 images. We show transformation vectors between correspondences in Fig. 10. Table 4. demonstrates the performance of DSMM on T00 and T50 of each subject.

**Table 4:** Mean 3D Euclidean magnitude distance (unit: mm) between correspondence of T00 and T50 by using DSMM.

	1	2	3	4	5	6	7	8	9	10
before	3.89 ± 2.78	4.34 ± 3.90	6.94 ± 4.05	9.83 ± 4.86	7.48 ± 5.51	10.89 ± 6.97	11.03 ± 7.43	15.00 ± 9.01	7.92 ± 3.98	7.30 ± 6.35
after	0.05 ± 0.03	0.04 ± 0.03	0.03 ± 0.02	0.04 ± 0.02	0.09 ± 0.04	0.28 ± 0.06	0.05 ± 0.02	0.36 ± 0.13	0.03 ± 0.02	0.04 ± 0.03

**Figure 11:**
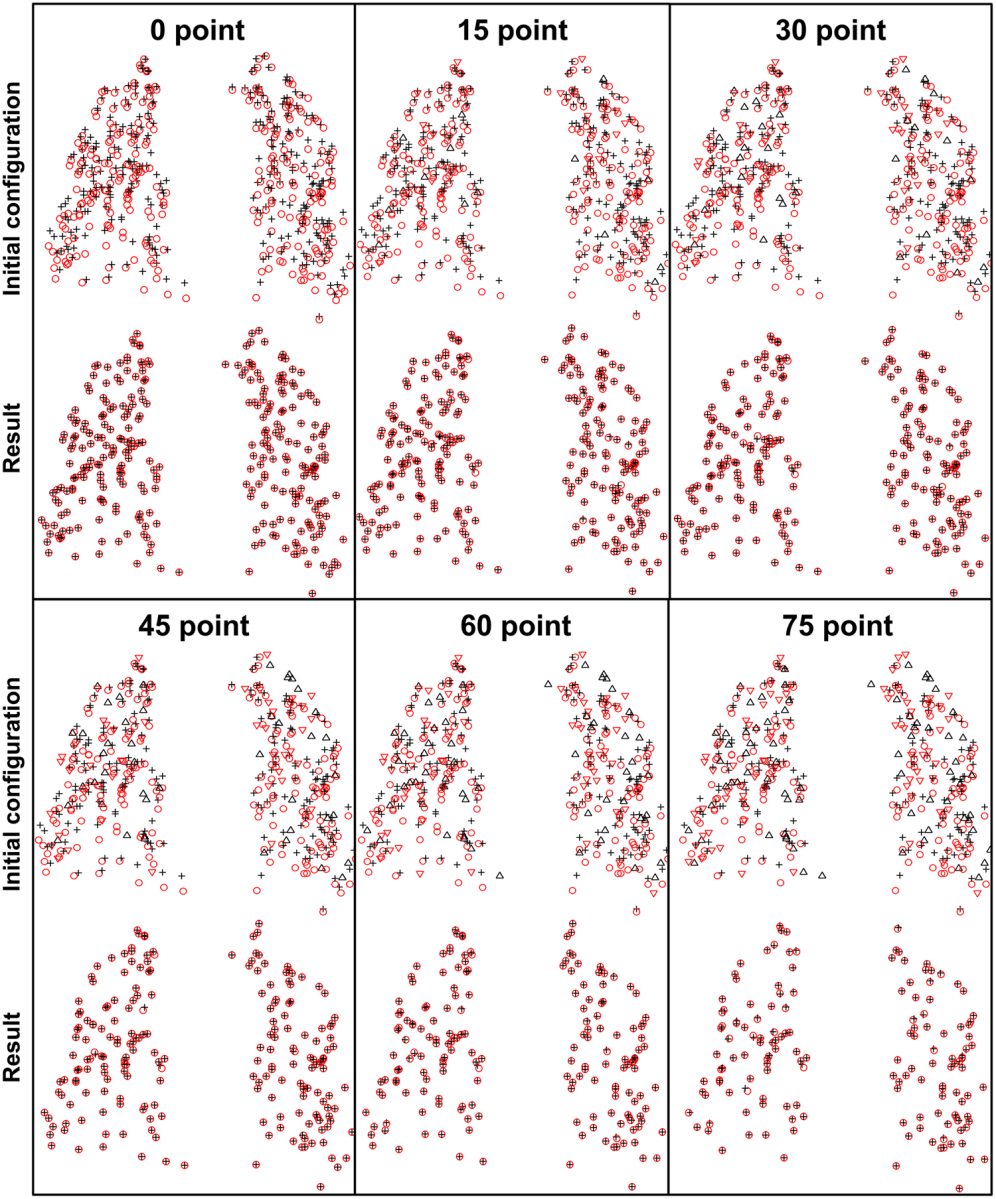
Performance of DSMM on the incomplete 3D lung data pairs identified from 4D CT images. We randomly remove increasing number of points both in the target point set and the template point set, which breaks up the one-to-one correspondence between the given data. For clarity, the red “∇” denote the missing correspondences, whose correspondences are removed in the template set, and the black “∆” denotes the missing correspondences in the template set. Our method shows its stable performance on the missing correspondences and occlusion. The top image in the each subfigure shows the initial configuration of incomplete data, and the bottom ones show the result of our method.

**Figure 12:**
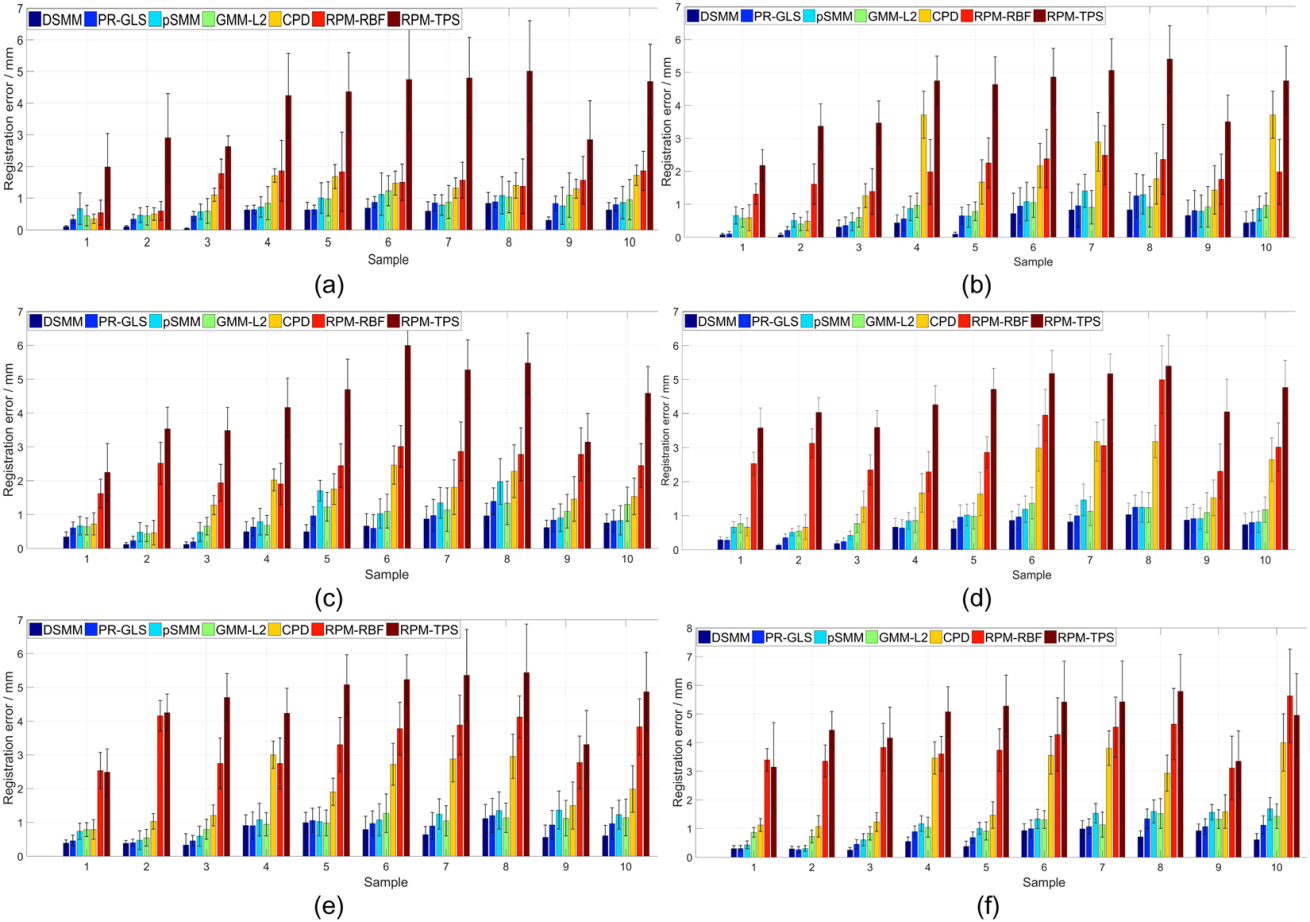
Quantitative evaluations of the non-rigid point set registration algorithms on the incomplete 3D lung data pairs of 4D CT images. (**a**) complete data, (**b**) 15 points removed, (**c**) 30 points removed, (**d**) 45 points removed, (**e**) 60 points removed, (**f**) 75 points removed.

We further test the ability of our algorithm to handle outliers and missing correspondences in the subsequent evaluation on the point sets from 4D CT images. In order to break up the one-to-one correspondence between the given point sets and add missing correspondences, we randomly delete the increasing number of points both in the target point sets and template points sets, as shown in the top row of Fig. 11. In the first subfigure, we do not delete any point, while in other subfigures, we respectively remove 15, 30, 45, 60, and 75 points both in the target set and the template set, which means only 270, 240, 210, 180, and 150 correspondences existing in figure (b)~(f). In order to explicitly reveal the outliers, we use red “∇” for denoting the outliers in the target set, whose correspondences having been removed in the template sets, and use black “∆” for denoting the outliers in the template set. Subsequently, we test DSMM and other algorithms on these pairs of incomplete samples. Figure 11 shows the performance of our method on these incomplete data. For clarity, we only show the correspondences in the result subfigures, which clearly shows that only few points diverge from the ground truth even though 75 points are removed in the data sets. In the evident from Fig. 11, our method shows its excellent performance in the presence of significant amounts of missing correspondences and outliers due to the local spatial representations and the prior probability modeling of each component in the mixture model. Figure 12(a–e) respectively show the mean 3D Euclidean magnitude distance between correspondences for different algorithms on the incomplete data sets, which indicates the statistical accuracy and robustness of our method.

**Figure 13:**
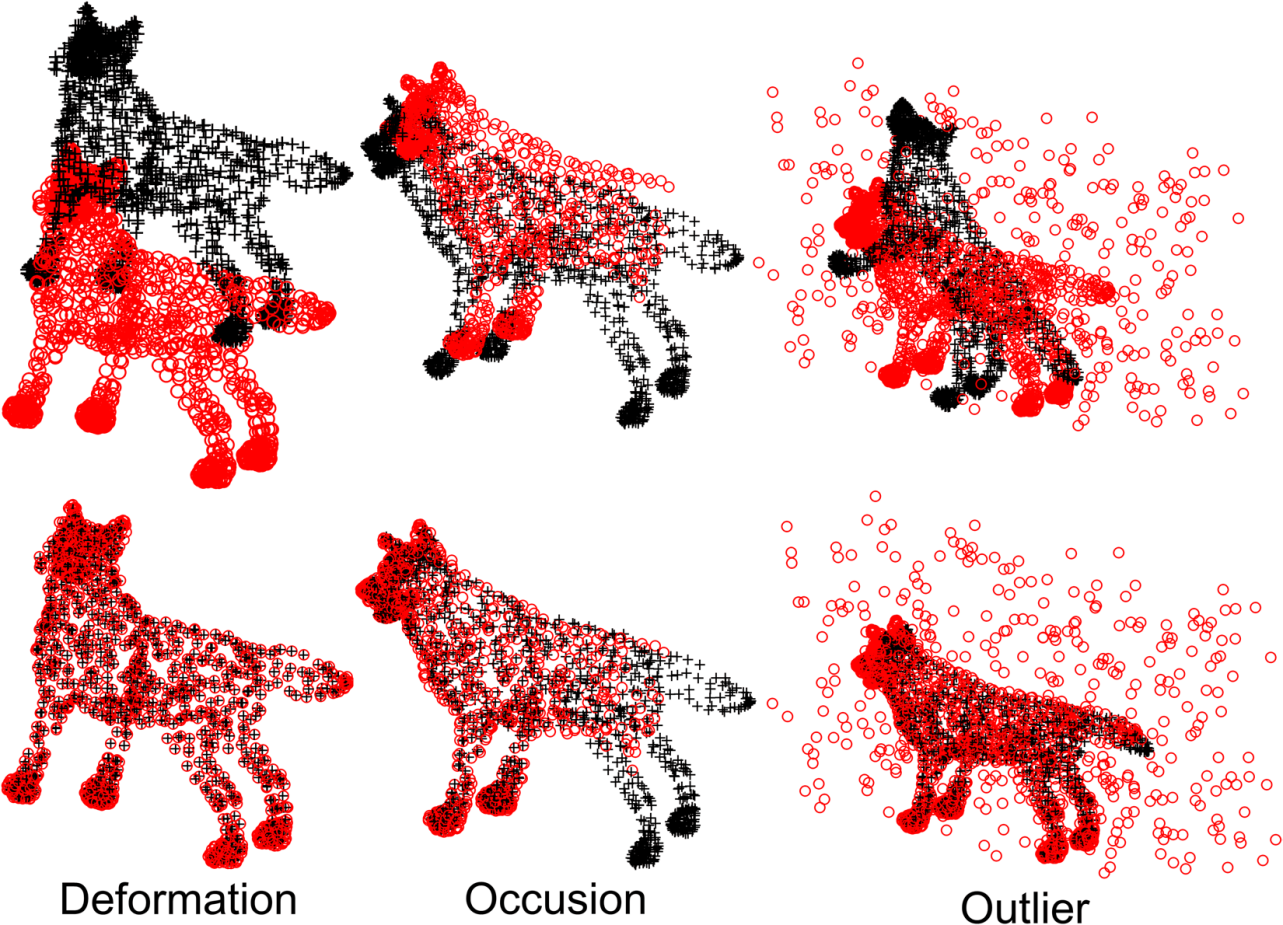
Preformation of DSMM on 3D wolf shapes with deformation, occlusion and outliers. We align the template points (black “+”) to the target points (red “o”) For each sample, the figures in the top row show template points and the target data before registration, and the figures in bottom show the registration results.

**Figure 14:**
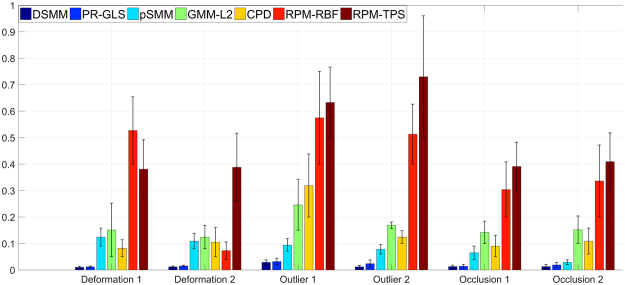
Performance of DSMM and other methods on wolf data.

Finally, we conduct the last quantitative experiment for matching 3D surface-like “wolf” shapes. Each point set typically contains about 5000 points, and there is absence between template point and target points. We show only 1600 points in the top row of Fig. 13 for clarity. In order to evaluate the robustness of DSMM on occlusion and outliers, we remove about 25 percentage of total number of points for representing occlusion, and add about 25 percentage of total number of points for representing outliers, which are respectively shown in the middle and right columns. The figures in top row of Fig. 13 show the superimposed points before registration, and the bottom figures show the matching results of DSMM. Figure 14 shows quantitative comparisons of DSMM and other competitive methods on wolf data, where DSMM performs the best results on ideal data and degeneration data.

## Discussion

Point set registration is a key problem in various applications. We focus on the model of point set registration which is a core point that has been received sustaining attentions in the recent years. In this work, we introduce a SMM-based non-rigid point set registration approach, named DSMM, which models the prior probabilities by using Dirichlet distribution and Dirichlet law. The main motion of our method is that we want to use a Bayesian framework to estimate the prior probabilities since the existing methods estimate them via a least-square method, which is a well-known method lack of robustness. Fortunately, Dirichlet distribution and its mixture models are fully Bayesian framework, which could automatically determine the model complexity (in terms of the total number of necessary mixture components) based on the data, not depend on any prior knowledge. Concretely speaking, we firstly consider the non-rigid point set registration as a probability density estimation, where one point set is represented as Student’s-t mixture model centroids, the other one is represented as data set. The main advantage of multivariate Student’s-t distribution is that it is heavily tailed than the Gaussian distribution, hence it is more robust against degradations than GMM. Secondly, we explicitly exploit Dirichlet distribution and Dirichlet law to incorporate the local spatial representation in the given point sets. We later assign various prior probability values of prior distribution depending on the input point sets, instead of the same value to all points, leading DSMM be more accurate than other existing algorithms. Thirdly, we formulate the SMM as an infinite scaled GMM integral form in order to obtain closed-form solutions. Subsequently, we iteratively fit the SMM centroids to the data set by using EM framework and estimate the posterior probabilities of centroids, which provides correspondence probabilities between the target point set and the template set. Finally, we calculate all registration parameters and transformation via the EM framework. We perform qualitative and quantitative evaluations for DSMM on various shapes. These evaluations intuitively indicate the favorable performance of DSMM.

## Conclusion

We have tested DSMM on various shape (2D contour-like, 3D cloud-like and 3D surface-like) point sets, and compared it with pSMM, PR-GLS, GMM-L2, CPD, RBF-RPM, and TPS-RPM. The results demonstrate that DSMM is robust against significant amount of missing correspondences and outliers, and is more accurate and robust than the other existing non-rigid point set registration methods.
